# Genetic diagnosis of Alport syndrome in 16 Chinese families

**DOI:** 10.1002/mgg3.2406

**Published:** 2024-03-03

**Authors:** Tangli Xiao, Jun Zhang, Li Liu, Bo Zhang

**Affiliations:** ^1^ Department of Nephrology, the Key Laboratory for the Prevention and Treatment of Chronic Kidney Disease of Chongqing, Chongqing Clinical Research Center of Kidney and Urology Diseases Xinqiao Hospital, Army Medical University (Third Military Medical University) Chongqing P.R. China

**Keywords:** Alport syndrome, *COL4A3*, *COL4A4*, *COL4A5*, variants

## Abstract

**Background:**

Alport syndrome (AS) is a genetically heterogeneous disorder resulting from mutations in the collagen IV genes *COL4A3, COL4A4*, and *COL4A5*. The genetic diagnosis of AS is very important to make precise diagnosis and achieve optimal outcomes.

**Methods:**

In this study, 16 Chinese families with suspected AS were recruited after pedigree analysis, and the clinical presentations were analyzed by a nephrologist. The genetic diagnosis was performed by whole‐exome sequencing (WES) and the disease‐causing variants were confirmed by Sanger sequencing.

**Results:**

The cohort of probands included seven men and nine women, with a mean age of 19.9 years. Pathological analysis showed slight‐to‐moderate mesangial proliferation, and thin basement membrane was the main findings. Pathogenic variants were revealed by WES in each family, and the co‐segregation with renal presentation was confirmed by PCR. In addition, RT‐PCR analysis showed that the intronic variant led to aberrant splicing.

**Conclusion:**

Our findings expand the spectrum of AS gene variation, which will inform genetic diagnosis and add to the theoretical basis for the prevention of AS.

## Abstract 2

Pathogenic variants were revealed by WES in each family, and the co‐segregation with renal presentation was confirmed by PCR. Our findings expand the spectrum of gene variation.

## 1 INTRODUCTION

Alport syndrome (AS) is an inherited glomerular disease that is the second most common hereditary kidney disease leading to renal failure after autosomal dominant polycystic kidney disease (Savige et al., 2013). AS is characterized by hematuria, proteinuria, and progressive renal failure, and some patients are accompanied by extrarenal manifestations (Rheault et al., 2020). The mutations of those genes *COL4A3/4/5* (OMIM 303630, 120070, and 120131) are responsible for the disease. AS can be transmitted as an X‐linked, autosomal recessive, autosomal dominant disorder, or digenic inheritance (Kashtan, 2021). Therefore, AS has strong genetic heterogeneity.

Traditionally, the clinical diagnosis of AS is based on histopathological findings, especially by electron microscopy. Most importantly, a positive family history of renal failure helps to make a diagnosis decision. In addition, the extra‐renal manifestations including sensorineural hearing loss and ocular abnormality are also helpful. However, the electron microscopic findings of renal biopsy may be atypical for those young patients, who are often misdiagnosed. Thin basement membrane (TBM) is often found among AS patients, but this feature is not specific enough to distinguish AS patients from other types of renal diseases. In addition, mutations of other genes can also lead to TBM. For example, a recent report showed that unusual TBM was found in a patient with nail‐patella syndrome (NPS), which was caused by a heterozygous variant in the LMX1B gene (Morimoto et al., 2021). Moreover, patients with primary diagnosis of familial focal segmental glomerulosclerosis (FSGS) have been proved to have variants in *COL4* genes (Papazachariou et al., 2017). Therefore, genetic testing is the best way to make a precise diagnosis.

Recently, the advances in comprehensive genetic analysis have enabled genetic testing to be performed for the diagnosis of AS first‐line diagnosis (Caliskan & Lentine, 2022). Genetic testing is much more sensitive and specific to the diagnosis of Alport syndrome, and can provide predictive information about disease severity and prognosis (Kashtan & Gross, 2021). It is also very important to aid treatment decisions and to give genetic counseling for patient's family. Recent expert guidelines recommend genetic testing for the diagnosis of AS (Savige, Mack, et al., 2021). The *COL4A3, COL4A4*, and *COL4A5* genes consist of approximately 50 exons, and the main strategy for gene screening in AS has become targeted sequencing, which captures all exons and exon‐intron boundaries of all three genes, along with comprehensive sequencing analysis by NGS (Nozu et al., 2019). To date, more than 3000 different pathogenic variants in the *COL4A3, COL4A4*, and *COL4A5* genes are recorded (Weber et al., 2016). However, it appears that many possible variants of these genes are still unknown.

Although genetic testing is an accurate method to make a final diagnosis, it is hard to pick up those patients for genetic testing. Considering the expense of genetic testing, a cohort of suspected AS patients were subject to genetic testing in this study, which was mainly based on the clinical presentation. Here, we report the results of mutational analysis and the genotype–phenotype correlations in the cohort of patients.

## 2 SUBJECTS AND METHODS

We report the genetic diagnosis of autosomal dominant and X‐linked Alport Syndrome in 16 Chinese families. Further details of patient recruitment, medical history, pedigree, and methodology are provided in Supporting Information (Appendix S1 and Figure S1).

## 3 RESULTS

### 3.1 Demographics and clinical characteristics

All of the participants involved in the study were of Han ethnicity. Dominant inheritance was confirmed by pedigree analysis. The clinical and biochemical characteristics of probands were displayed in Table 1. The cohort included seven men and nine women, with a mean age of 19.9 years (range = 5–35 years old). Their first renal presentation was either microscopic hematuria or macroscopic hematuria. Most cases had proteinuria when the probands were hospitalized except P4, P12, and P13. Most probands had normal renal function except P8, P9, P10, and P11, and two probands had ERSD at diagnosis. Notably, five probands had been found with unilateral or bilateral kidney cysts, but no liver cysts were detected. Although extrarenal presentations are common in AS patients, only three probands showed extrarenal features of hearing and visual impairment.

**TABLE 1:** Clinical characteristics.

Family ID	Gender	Age^a^	Hematuria	Proteinuria	Serum creatinine	Renal cysts	Extrarenal features
P01	F	35	3+	Trace	67.4	ND	Negative
P02	F	26	3+	3+	69.5	ND	Negative
P03	F	30	+	1+	59.1	Bilateral	Negative
P04	M	15	3+/	−	67.7	ND	Negative
P05	F	11	3+	3+	44.1	ND	Hearing and visual impairment
P06	F	25	3+	3+	83.5	ND	Negative
P07	F	22	2+	2+	56.7	ND	ND
P08	M	16	3+	3+	3512.0	Left	Negative
P09	M	15	2+	2+	1254.0	ND	Negative
P10	M	12	3+	3+	644.0	ND	Negative
P11	F	5	3+	3+	198.3	ND	Hearing and visual impairment
P12	M	12	+	−	82.4	Right	Negative
P13	F	22		−	115	Bilateral	Negative
P14	F	26	3+	3+	86.1	Left	Negative
P15	M	16	3+	Trace	108.6	ND	Visual impairment
P16	M	31	3+	2+	46.7	ND	Negative

^a^ Age at diagnosis.

### 3.2 Pathological findings and treatment

Among these families, 16 probands had renal biopsy, and the main findings were shown in Table S1. Most cases showed slight to moderate mesangial proliferation (MP). In addition, ischemic sclerosis (IS) or FSGS was detected in half of these probands by light Microscopy. Immunofluorescence staining revealed that IgM or IgA deposits were positive in one patient. Electron microscopy showed thin basement membrane (TBM) was the most frequently observed feature, followed by splitting and lamellation of the GBM. Moreover, basket weave change of GBM was detected in two cases.

Treatments of AS patients were based on their clinical characteristics. Most cases received RAAS inhibitors (RAASI), while others received hormone and calcineurin inhibitors (CNI). The renal function of most cases declined gradually, with two cases deteriorated to ERSD.

### 3.3 Whole‐exome sequencing

**FIGURE 1:**
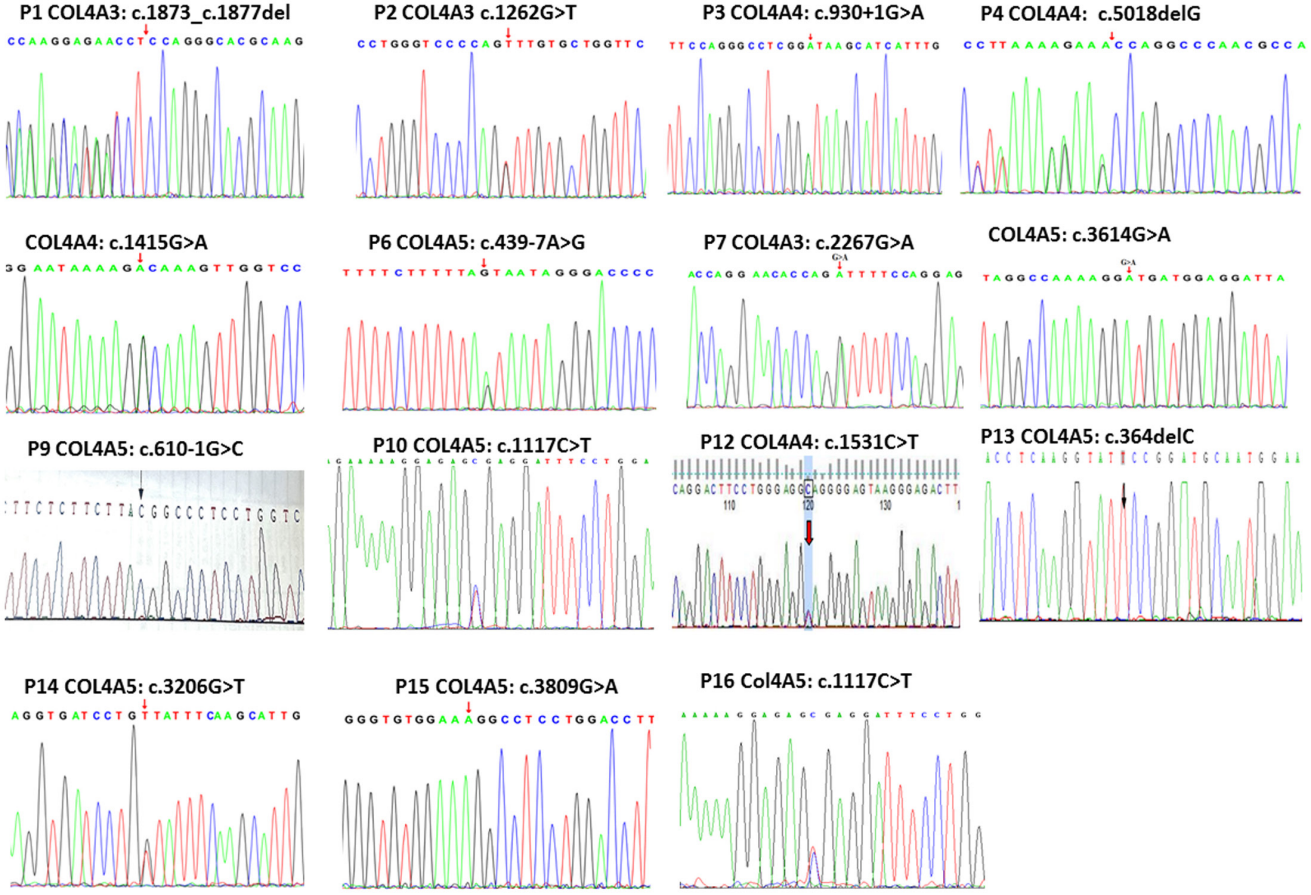
Identified variants were confirmed by Sanger sequencing.

Whole‐exome sequencing was performed to identify the potential disease‐causing variants of each family. After filtering against several databases, including dbSNP and the 1000 Genome Project, candidate variants were successfully identified in each family. The detected variants were shown in Table 2, and confirmed by Sanger sequencing (Figure 1). Totally, 15 different variants were identified, including six missense variants, four deletion variants, two stop gain variants, and three splicing variants in *COL4A3, COL4A4*, and *COL4A5* genes. Six variants were previously reported to be causative mutations for AS in other studies, while the others were novel in this study. PCR‐based sequencing in survival patients confirmed that each variant was co‐segregated with the renal presentation, which suggested a disease‐causing variant of each family (Figure S1).

**TABLE 2:** Variants summary.

Family ID	Gene	Nucleotide change	Amino acid change	Variant type	Novelty
P1	COL4A3	NM_000091 c.1873_c.1877del	p. G625Pfs*18	Deletion, het	Novel
P2	COL4A3	NM_000091 c.1262G>T	p.G421V	Missense, het	Novel
P3	COL4A4	NM_000092 c.930+1G>A	–	Splicing, het	Reported
P4	COL4A4	NM_000092 c.5018delG	p.S1673Tfs*15	Deletion, het	Novel
P5	COL4A4	NM_000092 c.1415G>A	p.G472D	Missense, het	Reported
P6	COL4A5	NM_000495 c.439‐7A>G	p.G147X	Splicing, het	Novel
P7	COL4A3	NM_000091 c.2267G>A	p.G756D	Missense, het	Novel
P8	COL4A5	NM_000495 c.3614G>A	p.G1205D	Missense, het	Reported
P9	COL4A5	NM_000495 c.610‐1G>C	–	Splicing, het	Reported
P10	COL4A5	NM_000495 c.1117C>T	p.R373X	Stop gain, het	Reported
P11	COL4A3	NM_000091 loss1(Exon:26–30)	–	Deletion, het	Novel
P12	COL4A4	NM_000092 c.1531C>T	p.Gln511X	Stop gain, het	Novel
P13	COL4A5	NM_000495 c.364delC	p.P123Dfs*30	Deletion, het	Novel
P14	COL4A5	NM_000495 c.3206G>T	p.G1069V	Missense, het	Reported
P15	COL4A5	NM_000495 c.3124G>A	p.G1042R	Missense, het	Novel
P16	COL4A5	NM_000495 c.1117C>T	p.R373X	Stop gain, het	Reported

Abbreviation: Het, heterozygous.

Among these variants, a 7.6‐kb deletion of *COL4A3* gene was identified in Family 11. This deletion located on chr2:228,137,664‐228,145, 306, spanning exon 26–30, and the copy number of exon 26, 28, and 30 of *COL4A3* gene was confirmed by Real‐time PCR analysis (Figure S2). Both the proband and her mother were heterozygous. Notably, the mother had no conscious symptoms, until microscopic hematuria was detected according to the advice.

### 3.4 Functional analysis of intronic variants

Among the identified intronic variants, two variants located at the conservative splicing sites. Mutations in the GT‐AG box are expected to be associated with splicing errors. However, the functions of the additional intronic variant needed to be verified. For this end, the effect on RNA splicing of intronic variant was predicted by the NetGene2 tools. As expected, analysis of NetGene2 showed that this substitution could lead to the generation of a novel splicing acceptor site (Figure S3). In order to confirm the splicing error, subcutaneous adipose tissue was obtained from the proband, and total RNA was isolated and detected by RT‐PCR. Subsequent Sanger sequencing revealed that an additional fragment was confirmed, which would lead to reading frame shift (Figure S3).

## 4 DISCUSSION

In the current study, we used WES for genetic diagnosis of 16 AS families. Fifteen variants in *COL4A3, COL4A4*, and *COL4A5* genes were found, which enlarged the variant spectrum in the Han Chinese population.

AS is thought to be a rare disorder, but its prevalence might be underestimated. According to a recent study (Gibson et al., 2021), the predicted pathogenic *COL4A5* variants were found in at least one in 2320 individuals and the predicted pathogenic heterozygous *COL4A3* and *COL4A4* variants affected one in 106 of the population, although the clinical presentation is not recorded as that high. Therefore, AS is thought to be the most common inherited kidney disease, even more prevalent than autosomal dominant polycystic kidney disease. Clinical features of AS patients range from isolated hematuria to kidney failure, as well as extrarenal manifestations. Notably, three probands were diagnosed earlier than their parents, whose renal presentations were just microscopic hematuria. Their renal presentations were found according to the nephrologist's advice. This observation indicated a huge inter‐ and intrafamilial variability of renal presentations among AS patients. In the current study, five probands had been found with unilateral or bilateral kidney cysts. Two patients had bilateral well‐formed renal cysts, and 3 patients had unilateral renal cysts, all of which were single, not multiple. This is different from that in polycystic kidney disease, which is mainly caused by mutations of PKD1 or PKD2. Interestingly, renal cysts of AS patients were also reported in other studies (Gulati et al., 2019; Savige, Storey, et al., 2021).

According to the guidelines for genetic testing and management of AS, experts now advocate genetic testing for persistent hematuria, even when a heterozygous pathogenic *COL4A3* or *COL4A4* is suspected, and cascade testing of their first‐degree family members because of their risk of impaired kidney function (Savige et al., 2022). Compared with those gene‐panel designs, WES helps to identify the potential variants contributing to the phenotypes (Bullich et al., 2018). In fact, we did find other disease‐causing variant (*GJB2*, NM_004004 c.109G>A) in Family P5. The homozygous variant also can lead to hearing loss. Thus, the role of this variant cannot be ruled out. Besides, WES also helps to identify the modifier genes of AS. Recently, Takemon Y et al. identified several loci associated with the variation in albuminuria and GFR in a mouse model, including a locus on the X chromosome associated with X inactivation and a locus on chromosome 2 containing *Fmn1* (Takemon et al., 2021). Therefore, genetic analysis based on WES of AS patients will shed light on the full understanding of the correlation of genotypes and phenotypes.

Among these AS families, renal failure was most frequently observed in patients with variants of *COL4A5* gene. In the eight families with variants of *COL4A5* gene, there were eight patients who died of ERSD, who were presumed to have pathogenic variants. In addition, four patients had ERSD. However, patients with heterozygous variants of *COL4A3* or *COL4A4* had better renal outcomes. This observation was consistent with previous reports (Savige, 2022). However, heterozygous subjects with pathogenic variants of *COL4A3/A4* gene are not expected to live a healthy life. Moreover, reports have shown that some patients with a primary diagnosis of familial FSGS proved to have variants in *COL4A3* or *COL4A4* genes (Deltas et al., 2015; Pierides et al., 2009). In this report, the proband of P11 had a pathogenic variant of *COL4A3* gene, and had presentations of chronic renal failure and FSGS. As our samples are rather small, it is hard to draw a conclusion of the correlation of genotypes and phenotypes.

In current study, eight variants were never reported. This suggested that the mutation spectrum of AS was very broad. Notably, three variants involved in RNA splicing were found. Two variants located at the conservational splicing sites. One variant was adjacent to the splicing sites. Actually, it is difficult to distinguish the intronic variants leading to splicing errors from those harmless polymorphisms. To date, there are several in silico approaches to assess the function of intronic variants, but functional analysis is needed to confirm the results in silico (Jian et al., 2014). The splicing errors induced by intronic variants can also be analyzed by in vivo assay, if possible. In family P6, we confirmed the splicing error by analyzing the RT‐PCR products, which was similar to our previous report (Wu et al., 2021). Therefore, the intronic variants should be fully investigated, especially for those adjacent to the conservational splicing sites.

In conclusion, the pathogenic intronic variants in *COL4A3*, *COL4A4*, and *COL4A5* genes were identified by WES in Chinese AS families. Our findings enlarged the mutational spectrum of AS. Identification of these pathogenic variants helps to understand the relationship between phenotypes and genotypes of AS.

## AUTHOR CONTRIBUTIONS

Bo Zhang conceived and designed research; Bo Zhang and Tangli Xiao performed the genetic investigations, Tangli Xiao, Jun Zhang and Li Liu collected patient data, Tangli Xiao drafted manuscript, Bo Zhang revised manuscript. All authors read and approved the final manuscript.

## CONFLICT OF INTEREST STATEMENT

The authors have no conflicts of interest to declare.

## ETHICS STATEMENT

Written informed consents to participate in the study were obtained from the patients and their parents. The present study was ethically approved by the Ethics Committee of Xinqiao Hospital at Army Medical University (Chongqing, China). All procedures were in accordance with the ethical standards of the responsible committee on human experimentation (institutional and national) and with the Helsinki Declaration.

## Supporting information


**Appendix S1.**.

**Figure S1.**.

**Figure S2.**.

**Figure S3.**.

**Table S1.**.

## Data Availability

All the data supporting the findings of this study are available from the corresponding author upon reasonable request.
