# Supplementary material for: Genetic diagnosis of Alport syndrome in 16 Chinese families
Source: Mol Genet Genomic Med. 2024 Mar 3;12(3):e2406. doi: 10.1002/mgg3.2406 (PMC10910213; doi:10.1002/mgg3.2406)
Supplement: Supplementary file 1 — Appendix S1.. [file MGG3-12-e2406-s001.docx]

**Genetic diagnosis of Alport Syndrome in 16 Chinese families**

Tangli Xiao, Jun Zhang, Li Liu, Bo Zhang

**Ethical Compliance**

The study was approved by the ethical committee of Xinqiao Hospital (No. 2020-147), and was in accordance with the principles of the Declaration of Helsinki. All participants in this study signed informed consent. Written informed consents from individuals under 18 years old were obtained from their parents.

**Recruitment of patients**

The clinical diagnoses of AS individuals from the department of nephrology at Xinqiao Hospital, Army Medical University were made by a nephrologist based on clinical manifestations, laboratory analysis, such as hematuria, proteinuria and creatinine levels, and a family history of persistent hematuria or AS. The diagnostic criteria for AS include: (1) hematuria, with or without proteinuria; (2) a family history of persistent hematuria, AS or ERSD patients; (3) Consent for genetic diagnosis by WES. Kidney histopathological data collection included light microscopy, immunofluorescence, and electron microscopy examination of biopsy tissues. All individuals with high-grade suspicion for AS provided a blood sample DNA extraction.

**Genomic DNA preparation**

Blood samples (1~2 mL) were collected from the probands and their family members. Genomic DNA was extracted using a QIAamp DNA Blood Mini Kit (Qiagen, Germany), according to the manufacturer’s instructions.

**Whole exome sequencing and Sanger sequencing**

The DNA samples from probands and their parents of each family were investigated by the whole exome sequencing (WES) for potential responsible variants, according to previous description [13]. Candidate pathogenic variants were validated by polymerase chain reaction (PCR) and Sanger sequencing in affected subjects of each family to explore the co-segregation of genotype and phenotype.

**Bioinformatics analysis**

To detect the potential pathogenic variants of the probands, clean reads were aligned against the human genome reference, and single nucleotide variants (SNVs) and indels were identified using the SOAPsnp software and Samtools Indel Genotyper, respectively. All SNVs and indels were determined using the NCBI Database of Single Nucleotide Polymorphisms (dbSNP) and subsequent bioinformatics analysis was performed based on online tools including PolyPhen2 and SIFT. Variants are classified according to the American College of Medical Genetics and Genomics standards and guidelines [14]. For intronic variants, the splicing effect was predicted via the NetGene2 software [15] (http://www.cbs.dtu.dk/services/NetGene2/).

**In vivo assay of mRNA splicing**

Subcutaneous adipose tissue was obtained from the proband of family P6 and total RNA was isolated. RT-PCR was performed to amply a cDNA fragment spanning the variants. Briefly, total RNA was extracted by using an RNA Isolation Kit (TakaRa, Dalian, China) and was reverse-transcribed into cDNA. Then regular PCR was performed on a cycler with corresponding primers. The product of RT-PCR was confirmed by Sanger sequencing.

**Validation of the loss of *Col4A3* exon 26-30**

Realtime PCR with corresponding primers was performed to detect the copy number of the region, normalized by that of albumin (ALB) gene in human genome. Briefly, realtime PCR amplification was done with a 10-μL final reaction mixture consisting of 50ng genomic DNA, 0.1 μM of the each sense and antisense primers, and 1× PCR mixture with SYBR Green I (BioRad, China), using the CFX96 Real-Time System (Bio-Rad). Copy number was calculated according to the formula: 2^[(Ct _(Col4A3)_-Ct_(ALB)_) patient - (Ct _(Col4A3)_-Ct_(ALB)_) refer sample].

References

1. Zhang J, Dai LM, Li FR, et al. A Chinese family of autosomal recessive polycystic kidney disease identified by whole exome sequencing. Medicine (Baltimore) 99: e20413, 2020.

2. Richards S, Aziz N, Bale S, et al. Standards and guidelines for the interpretation of sequence variants: a joint consensus recommendation of the American College of Medical Genetics and Genomics and the Association for Molecular Pathology. Genet Med 17:405-424, 2015.

3. Hebsgaard SM, Korning PG, Tolstrup N, Engelbrecht J, Rouzé P, Brunak S. Splice site prediction in Arabidopsis thaliana pre-mRNA by combining local and global sequence information. Nucleic Acids Res 24:3439-3452, 1996.

Figure S1 The pedigrees of the families with dominant Alport Syndrome. Squares and circles indicate men and women, respectively. The dark symbols represent the affected members and the gray symbols represent the asymptomatic members. The arrow indicates the proband.

Figure S2 Copy number of *COL4A5* gene was determined by Quantitative PCR in family P11.

Figure S3 Function analysis of the intronic variant. A: Analysis using NetGene2 has shown that the variant COL4A5 c.439-7A>G generated the novel potential splicing acceptor site. B: RT-PCR product was directly sequenced. Red line indicated the additional fragment.
