# Supplementary figures and images for: Genetic diagnosis of Alport syndrome in 16 Chinese families
Source: Mol Genet Genomic Med. 2024 Mar 3;12(3):e2406. doi: 10.1002/mgg3.2406 (PMC10910213; doi:10.1002/mgg3.2406)

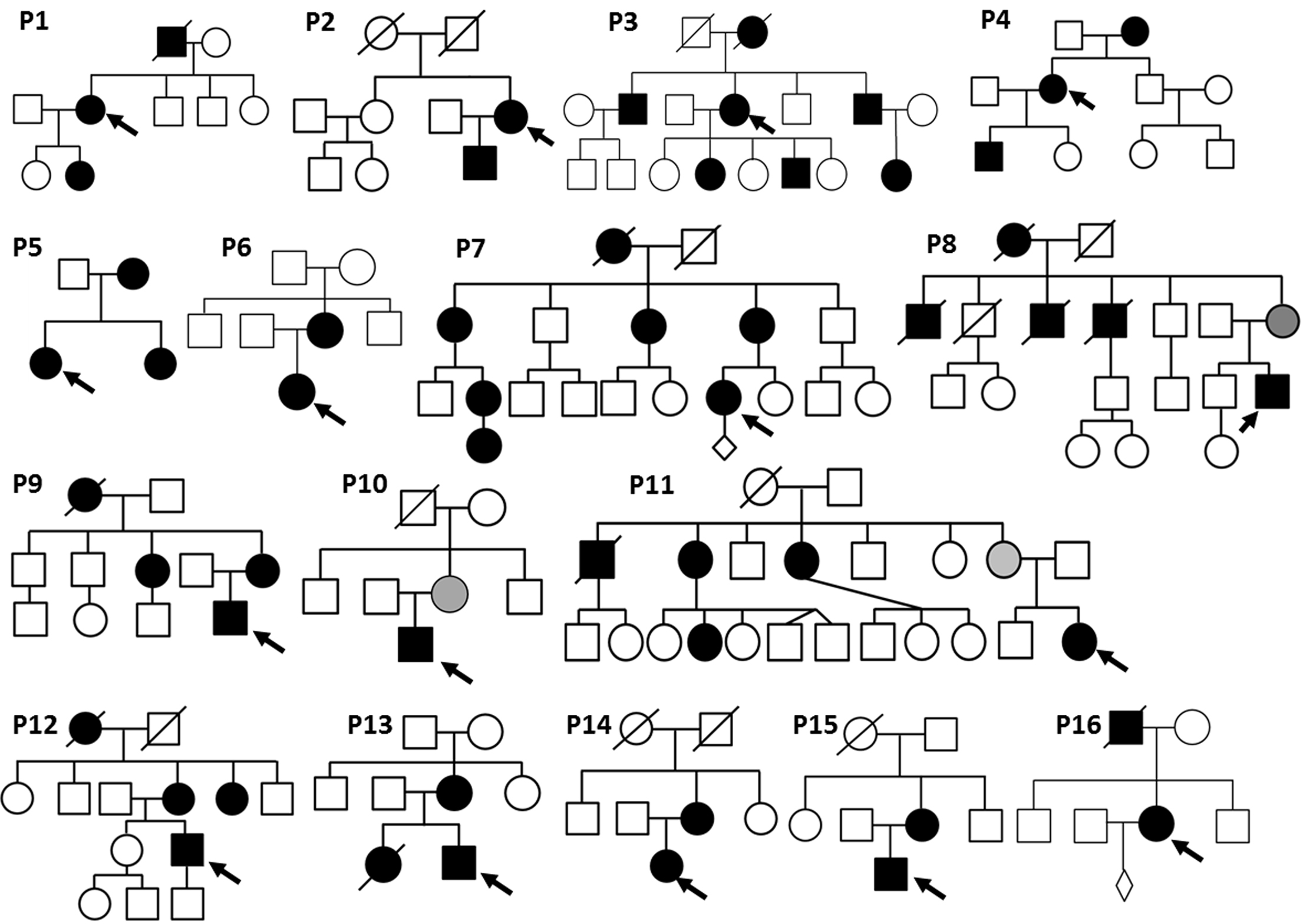

Supplement: Supplementary file 2 — Figure S1.. [file MGG3-12-e2406-s005.jpg]

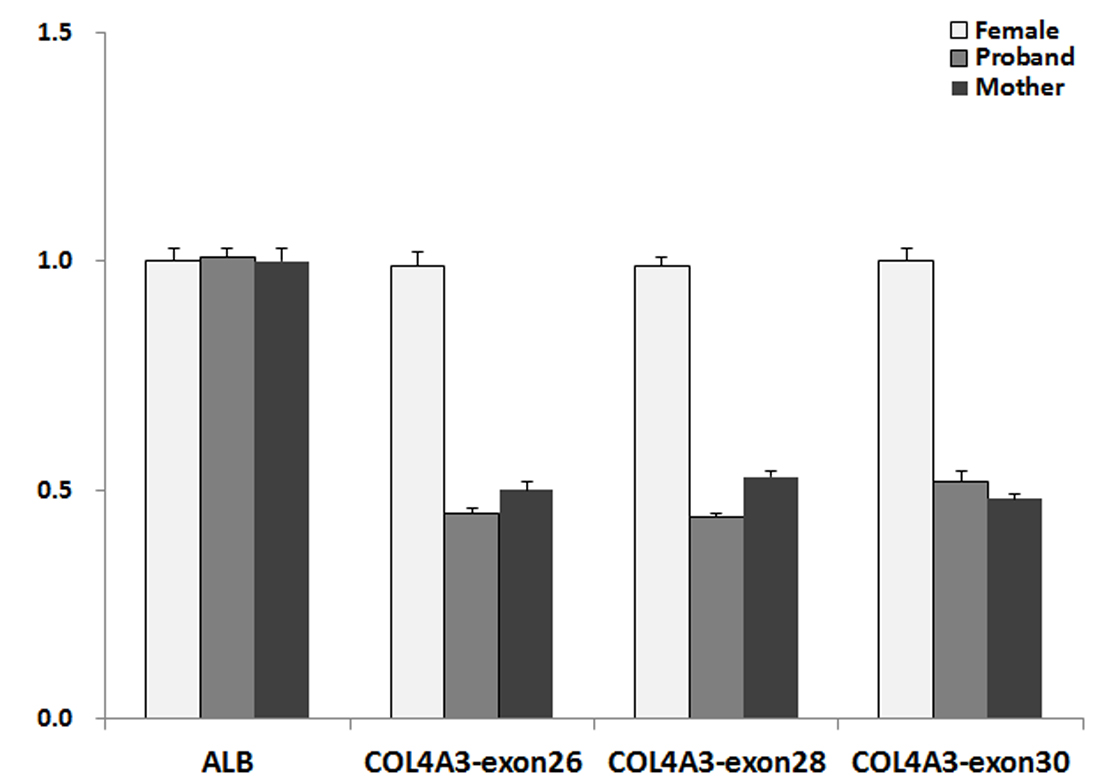

Supplement: Supplementary file 3 — Figure S2.. [file MGG3-12-e2406-s002.jpg]

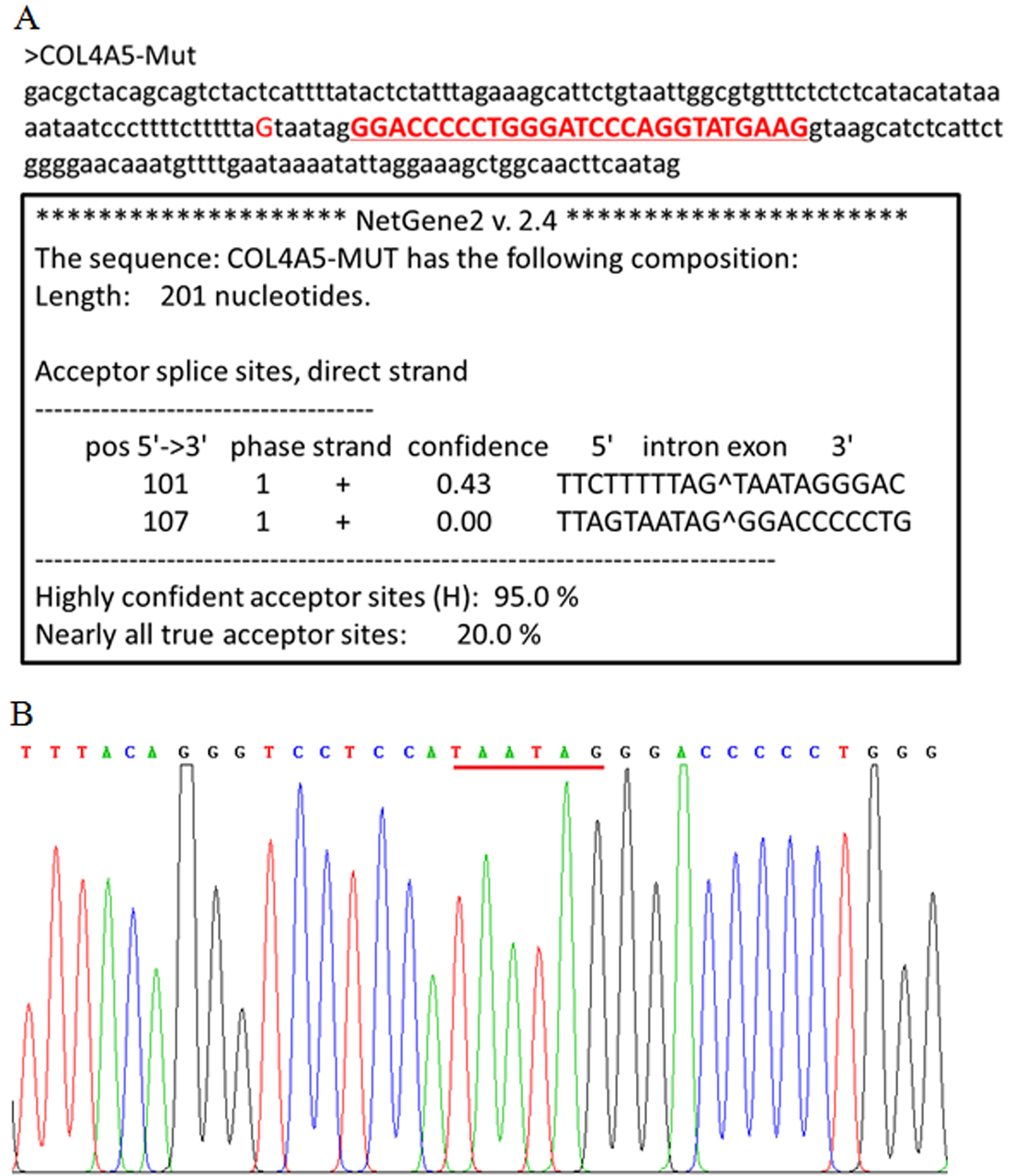

Supplement: Supplementary file 4 — Figure S3.. [file MGG3-12-e2406-s003.jpg]
