# Supplementary material for: Genetic diagnosis of Alport syndrome in 16 Chinese families
Source: Mol Genet Genomic Med. 2024 Mar 3;12(3):e2406. doi: 10.1002/mgg3.2406 (PMC10910213; doi:10.1002/mgg3.2406)
Supplement: Supplementary file 5 — Table S1.. [file MGG3-12-e2406-s004.docx]

Table S1 Renal biopsy and treatments of the probands

| ID | IF | Light Microscopy | Electron Microscopy | Treatment | Renal outcome |
| --- | --- | --- | --- | --- | --- |
| P01 | Negative | MP,FSGS | TBM | RASSI | Survival |
| P02 | Negative | MP | TBM | RASSI | Survival |
| P03 | Negative | MP,IS | TBM | RASSI | Survival |
| P04 | IgM | MP | TBM | Hormone/CNI | Survival |
| P05 | Negative | MP | TBM | Hormone/CNI | Survival |
| P06 | Negative | MP | TBM, SL | RASSI | Survival |
| P07 | Negative | MP,IS | TBM | RASSI | Survival |
| P08 | ND | ND | ND | KRT | ERSD |
| P09 | Negative | MP | TBM, SL, BWC | KRT | ERSD |
| P10 | Negative | MP,FSGS | TBM, SL | Hormone/CNI | ERSD |
| P11 | Negative | MP,FSGS | TBM, SL | RASSI | CRF |
| P12 | Negative | MP,IS | TBM | RASSI | Survival |
| P13 | Negative | MP,FSGS | TBM, SL | RASSI | CRF |
| P14 | Negative | MP | TBM, SL | RASSI | Survival |
| P15 | Negative | MP | TBM, SL | Hormone/CNI | CRF |
| P16 | IgA | MP | TBM, SL, BWC | RASSI | Survival |

IF: Immunofluorescence Staining; MP,Mesangial proliferation; IS:ischemic sclerosis; TBM: Thin basement membrane; SL: splitting and lamellation of the GBM; BWC: basket weave change; CRF: chronic renal failure.
